# Interplay between economic empowerment and sexual behaviour and practices of migrant workers within the context of HIV and AIDS in the Lesotho textile industry[Fn FN0001]

**DOI:** 10.1080/17290376.2014.976250

**Published:** 2014-11-10

**Authors:** Pius Tangwe Tanga, Magdaline Nji Tangwe

**Affiliations:** ^a^ PhD, is a professor and postgraduate coordinator affiliated to the Department of Social Work/Social Development, University of Fort Hare, PB X1314, Alice5700, South Africa; ^b^ PhD student, Faculty of Education, University of Fort Hare, PB X1314, Alice5700, South Africa

**Keywords:** economic empowerment, migrant workers, sexual behaviour and practices, HIV and AIDS, options and choices, autonomisation économique, travailleurs migrants, comportements et pratiques sexuels, VIH/SIDA, options et choix

## Abstract

Economic empowerment brings with it a wide range of consequences, both positive and negative. The objective of this paper was to examine the relationship between economic empowerment and the sexual behaviour and practices of migrant workers within the context of HIV and AIDS in the Lesotho textile industry. Data for this paper were extracted from the findings of a larger study which had been conducted concerning HIV and AIDS in the textile industry in Lesotho. Using in-depth interviews, data were collected from 40 participants who were purposively selected from five factories which had been chosen randomly. Empowerment theory was used as a lens to provide meanings for the experiences of the participants. The findings show that the participants were empowered only in certain respects in terms of Kabeer's empowerment model of ‘power to’ and ‘power within’, on one hand, and in terms of Malhotra's comprehensive empowerment framework at the household level, on the other, as being employed in the industry enabled them to participate in the economy. Employment in the sector provided the participants with the means to be able to acquire basic needs and the ability to participate in household decision-making: for the female participants, the ability to make independent sexual decisions was also enhanced. These improvements were greeted enthusiastically, particularly by the female participants, given their previously disadvantaged status as a result of coming from rural patriarchal villages with gender-defined hegemonic notions of respectability. The findings also indicate that environmental factors and others, such as meagre salaries, encouraged some of the female workers to engage in transactional sex, while some of the male participants tended to increase their sexual relationships as a result of acquiring employment and income from the industry. It is the contention of the authors of this study that true empowerment requires both vital resources and individual and collective participation, particularly for the women, who are more vulnerable than men. Finally, we conclude that the opportunities provided by economic empowerment have given the participants a new social meaning for their situation and an awareness about their place in power relations.

## Résumé

L'autonomisation économique apporte avec elle un large éventail de conséquences, à la fois positives et négatives. L'objectif de cette étude était d'examiner la relation entre l'autonomisation économique et les comportements et pratiques sexuels des travailleurs migrants dans le contexte du VIH/SIDA dans l'industrie textile du Lesotho. Les données de l'article ont été extraites des résultats d'une plus grande étude qui avait été menée concernant le VIH/SIDA dans l'industrie textile au Lesotho. Par le biais d'entrevues en profondeur, les données ont été recueillies auprès de 40 participants choisis à dessein, de cinq usines choisies au hasard. La théorie de l'autonomisation a été adoptée comme lentille pour analyser les expériences des participants. Les résultats ont montré que les participants n'ont été autorisés qu'à certains égards en termes de modèle d'autonomisation de «pouvoir de» et «pouvoir intérieur» préconisé par Kabeer, d'une part, et de cadre global de l'autonomisation au niveau des ménages selon Malhotra de l'autre part ; étant donné que le fait d’être employé dans l'industrie leur a permis de participer à l'économie. L'emploi dans le secteur a fourni aux participants les moyens d'être en mesure d'acquérir des besoins de base ainsi que la capacité de participer à la prise de décision des ménages. Pour les participants de sexe féminin, la capacité de prendre des décisions sexuelles indépendantes a également été améliorée. Ces améliorations ont été accueillies avec enthousiasme, en particulier par les participantes, compte tenu de leur statut précédent de défavorisées, étant originaires des villages patriarcales ruraux où des notions hégémoniques de respectabilité sont définies selon des critères de genre. Les résultats indiquent également que les facteurs environnementaux, ainsi que d'autres facteurs tels que les salaires maigres, encouragent certains des travailleurs féminins à avoir des relations sexuelles transactionnelles, tandis que certains participants masculins avaient tendance à augmenter leurs relations sexuelles à la suite de l'acquisition de l'emploi et des revenues de l'industrie. L'affirmation des auteurs de cette étude est que la véritable autonomisation requiert des ressources fondamentales et la participation individuelle et collective, en particulier pour les femmes, qui sont plus vulnérables que les hommes. Enfin, nous concluons que les possibilités offertes par l'autonomisation économique ont donné un nouveau sens aux participants par rapport à leur situation sociale, ainsi qu'une sensibilisation sur leur place dans les relations de pouvoir.

## 1. Introduction

There is limited research on the linkage between economic opportunities provided by the giant textile industry in Lesotho and the sexual behaviours and practices of rural and urban migrant workers within the context of the ravaging HIV and AIDS pandemic in the country, despite the long history of the disease. The history of HIV and AIDS dates back to 1986, when the first case was reported in Lesotho. Since then, the pandemic has continued to take its toll on the country, with an adult prevalence rate of 23.2%, the third highest in the world after Botswana and Swaziland (National AIDS Commission 2010; UNAIDS 2009). This prevalence rate constitutes a third of the country's population. The devastating pandemic has already resulted in a crisis in the form of 221,403 orphans (Bureau of Statistics 2007). Despite these hardships, the country's circumstances are, to a certain degree, ameliorated by its textile industry, which provides employment to the rural masses, particularly to poor women from the mountainous regions of the country. Employment provided by the textile industry helps to offset the effects of declining employment in the South African mining industry for citizens of Lesotho, which had provided the major source of income to their households in the form of remittances, which had contributed significantly to the Gross National Product (Tanga 2013). The textile industry has a workforce of approximately 80,000 and is the largest employer of labour in the economy (MFA Forum 2007). While serving as a pro-poor growth initiative, most of the employment provided by the textile industry takes the form of unskilled and semi-skilled low paying jobs (Bello, Tsikoane, Mochebelele, Tanga, Nchake, Makatjane, *et al*. 2009). In 2007 the industry employed approximately 47,040 workers, of whom 16,000 were HIV positive (Com Mark Trust 2007a, 2007b). The industry employs approximately 86% of the women in Lesotho and is the largest textile industry in Africa (Bennet 2006). The importance of the industry for the country and its economy and the negative impact of the pandemic on the sector warrant a study to establish the connection between economic empowerment and sexual behaviour and practices, in order to guide local and national responses to the pandemic.

It has been maintained that HIV and AIDS coincided with the changing political opportunities which encouraged initiatives for women by women's rights advocates (Bello *et al*. 2009). According to Braun and Dreiling (2010), HIV and AIDS have devastated the productive adult population, which had formerly migrated in order to find employment, both within Lesotho and in South Africa. As a result of the high rate of retrenchment of male miners from South Africa, women have become obliged to migrate from the rural to the urban areas in search of employment in the textile industry. Braun and Dreiling (2010) conclude that this migration pattern is a result of the high rates of poverty and unemployment, which are major factors in the spread of HIV. UNIADS (2005:39) defines vulnerability as the likelihood of exposing oneself to a number of factors in the external environment that are not under the control of the person or social group. Owing to the unequal gender relations and entrenched gender inequality in society, UNAIDS (2005) maintains that women and girls from poor socio-economic backgrounds have a greater risk of being vulnerable to HIV infection than their more affluent counterparts. In addition, the migration of workers from rural to urban areas has increased their vulnerability to HIV infection, owing to the possibility of their having sex with people other than their partners.

Social and political empowerment of the poor is increasingly seen as ‘the basis for an alternative grass-roots model of global political and economic development’ (Sadan 2004:770). According to Kalipeni and Zerai (2007), the vulnerability of women to HIV is exacerbated by widespread gender-based social inequalities. HIV transmission can be stemmed only when issues of vulnerability are addressed meaningfully. Economic deprivation is recognised as an important driver in the spread of HIV and risky sexual behaviour and, accordingly, it may be concluded that poverty compounds vulnerability to HIV and that its impact is felt more in urban than in rural areas (Dodoo, Zulu & Ezeh 2007). It is generally accepted that in Africa the interplay between gender and socio-economic factors contributes significantly to the vulnerability of both men and women to HIV. Gender-related cultural norms tend to create barriers to the full participation in and the ability to benefit from the productive economy for women, which in turn makes women dependent on male partners for important household decisions, which limits their ability to negotiate safer sex (Masanjala 2007). Many women who are on the verge of becoming destitute and who face unequal access to a means of earning a living are tempted to engage in transactional sex in order to survive (Camlin, Kwena, Dworkin, Cohen & Bukusi 2014; Hunter 2010; Masanjala 2007). The textile industry has the potential to become an important pro-poor initiative to enhance poor people's access to opportunities, security and empowerment, which could, in turn, foster economic growth and poverty reduction. This study will focus on the relationship between economic opportunities and the sexual behaviour and practices of workers against the background of the high rate of prevalence of HIV in the country.

### 1.1. Empowerment theory

We draw on the theory of empowerment to understand the link between migrant workers’ experiences of economic empowerment and their sexual behaviour within the context of the prevalence of HIV and AIDS. The freedom of the poor and the disadvantaged is curtailed by their voicelessness and powerlessness, both of which are related to the state and the economy. Powerlessness is also embedded in a culture of unequal institutional relations and gender inequalities (Sadan 2004). When people are disempowered, they feel a sense of powerlessness, which leads to a lack of feelings of self-worth, a tendency towards self-blame and dependence on social services for solutions to social problems. There are instances when limitations, such as poverty and a lack of education, can act as powerful factors inhibiting people's sense of personal power to the extent that they feel unable to act at all (Sadan 2004).

The concept of empowerment is difficult to define and it embodies a process which is becoming a tool for analysis. The process of empowerment is dynamic and multidimensional in nature, and various approaches and frameworks have been developed to assess it (Murthy 2002). Empowerment counters feelings of hopelessness and powerlessness, and emphasises personal ability to make and implement basic life decisions (Clarke 2000). Empowerment is essentially the process of gaining power in the ways described by many authors of works relevant to this study (Ginige & Richards 2012).

Many definitions of empowerment use terms such as control, self-reliance, the freedom to define, the ability to make one's own decisions, independence and self-sufficiency (Narayan 2002). The concept of empowerment may be applied at both the individual and the collective levels. Narayan (2002:xviii) broadly refers to empowerment as the expansion of freedom of choice and action in order to shape one's life. It implies control over resources and decisions. In a World Bank publication titled *Empowerment and poverty reduction: A sourcebook*, Narayan (2002:xviii) asserts that ‘Empowerment is the expansion of assets and capabilities of poor people to participate in, negotiate with, influence, control, and hold accountable institutions that affect their lives’. Kabeer (2001) defines empowerment as ‘the expansion in people's ability to make strategic life choices in a context where this ability was previously denied to them’ (cited in Malhotra 2003:3).

Empowerment is a process of transition from a state of powerlessness to a state of relative control over one's life, destiny, and environment. This transition can manifest itself in an improvement in the perceived ability to control, as well as in an improvement in the actual ability to control. (Sadan 2004:144)

In this sense, empowerment is the transition from a passive situation to one of more active control.

These definitions have features in common, in terms of the process and its outcomes. Empowerment theory states that actions and activities or structures may empower people and that each outcome of each process results in a level of being empowered (Zimmerman 1988). According to Ginige and Richards (2012), a thorough analysis of empowerment would require multiple levels of analysis. ‘Empowerment processes for individuals might include learning decision-making skills, managing resources and working with others. Possible outcomes for individuals feeling ‘empowered’ would be situation-specific perceived control, critical awareness, skills and proactive behaviours’ (Ginige & Richards 2012:3). Of importance to empowerment are resources and agency (e.g. control, awareness, voice and power), though the former are treated as catalysts for empowerment or as ‘enabling factors’ which help in the process of empowerment. Agency refers to the strategic formulation of choices and then to the controlling of resources and decisions which affect important life outcomes (Sadan 2004:3). Empowerment theory is based on the concept that individuals or groups should be able to gain a voice in the making of decisions which affect them and also in the social structures which either encourage or discourage their development. Accordingly, empowerment in research predicts and explains the behaviour of an individual or a group within social constraints and the amount of power that is obtained from the outcomes of decisions (Rappaport 2013).

As migrants from the rural areas are assumed to have been previously disadvantaged in many respects, socio-economic and political empowerment processes should help to enhance mental, spiritual and physical wellness and to ensure social justice. Our intention was to examine whether economic opportunities, possibly resulting in economic empowerment, have triggered a shift in the sexual behaviour and practices of textile workers within the context of the prevalence of HIV and AIDS in the country. A useful definition adopted for this study was provided by Lord and Hutchison (1993), in that it is limited to individual analysis, such as might be applied to the migrant workers in this study. They define empowerment ‘as processes whereby individuals achieve increasing control of various aspects of their lives and participate in the community with dignity’ (Lord & Hutchison 1993:4).

In order to explore the link between migrant workers’ economic empowerment and their sexual behaviour, we used Kabeer's empowerment model (Kabeer 2001). According to this model, for empowerment to occur, certain interventions must be made and these include the following: the expansion of the material alternatives from which to choose; the enhancement of the ability to make choices which are consequential and the creation of alternatives from which to choose, and to increase the capacity to make choices, which should result in the transformation of gender relations (Kabeer 2001). Although several other domains have been included in Kabeer's model of empowerment, this study confined itself to only two of them, namely ‘power to’ and ‘power within’. *Power to* ‘relates to having decision-making authority, power to solve problems and can be creative and enabling’, while *power within* ‘refers to self-confidence, self-awareness and assertiveness’, and also to how individuals can, through the analysis of the experience in how ‘power operates in their lives, gain the confidence to act, to influence and to change’ (Oxaal & Baden 1997:1).

We also made use of Malhotra's (2003) comprehensive framework of the dimensions of women's empowerment, which can be applied in various different settings and contexts. According to Malhotra (2003:5), there are six dimensions that run across household, community and broader arenas. These include the economic, familial and interpersonal, psychological, legal, sociocultural and political dimensions. For the purposes of this paper, only three of these dimensions have been investigated: the economic, familial and interpersonal and the psychological, and only at the household level, without considering the community and broader arenas, as we contend that empowerment begins at the household level before extending to the community and broader society (see Table 1). In this paper we have examined only individual empowerment, which can occur in a variety of circumstances and conditions, and not collective empowerment.

**Table 1.** Dimensions of empowerment as used in this paper.

Dimension	Household
Economic	Women's control over income; relative contribution to family support; access to and control of family resource
Familial/Interpersonal	Participation in domestic decision-making; control over sexual relations; ability to make decisions on the use of contraception; control over spouse selection
Psychological	Self-esteem; self-efficacy; psychological well-being

Source*:* Extracted from Malhotra (2003:5).

Although this paper concerns the empowerment of both men and women, it is necessary to highlight what constitutes empowerment for women, as they are always seen to constitute a vulnerable group needing particular attention. Although all the definitions and characteristics of empowerment described in this paper apply to both genders, women's empowerment encompasses unique additional elements because, as Malhotra (2003:2) cautions, women comprise a cross-cutting category of individuals which overlaps with other groups. Second, households and interfamilial relations are central features in women's disempowerment, which do not apply to other disadvantaged groups. Women's empowerment concerns more than financial gain alone: it strives to enable women to live lives characterised by well-being and dignity, based on equality, rights and justice. The Inter-American Development Bank (2010:3) defined women's empowerment in terms of ‘expanding the rights, resources, and capacity of women to make decisions and act independently in social, economic, and political spheres’. The UN (2001) defined women's empowerment in terms of five components:

women's sense of self-worth; their right to have and determine choices; their right to have access to opportunities and resources; their right to have the power to control their own lives, both within and outside the home, and their ability to influence the direction of social change to create a more just social and economic order, nationally and internationally.

This study was based on the recognition that the textile industry in Lesotho has enhanced the country's economic potential by contributing positively towards the economic empowerment of workers. On the other hand, the rapidly changing nature of the economy increasingly exposes workers to globalised consumer patterns, which leads to changes in sexual behaviour, which may, in turn, increase their vulnerability to HIV infection in the specific circumstances created by the pandemic in Lesotho.

### 1.2. Migration and risk of HIV infection

Insufficient attention has been paid to the relationship between urban poverty, especially poverty among rural–urban migrants, and risky sexual behaviour in Lesotho. There is rapid urbanisation, not only in Lesotho, but also in most of Africa, with a corresponding growth of impoverished urban slum settlements, which are characterised by highest rates of HIV in the world (Greif & Dodoo 2011). Dodoo, Zulu and Ezeh (2007) found that in Kenya poverty is significantly related to risky sexual behaviour and noted that although the poor are generally the most adversely affected in this regard, the urban poor were considerably more disadvantaged than their rural counterparts. Consequently, the urban poor are more vulnerable and more likely to engage in risky behaviour as a result of high unemployment, unstable wages, financial insecurity and the desperate need to have money. Roy, Anderson, Evans and Rahman (2010) found very high levels of pre- and extramarital sexual behaviour, including high levels of risky and unsafe sex, among rural–urban migrant taxi drivers in Dhaka, Bangladesh. A high incidence of risky sexual behaviour was found among men who were living apart from their spouses, and the frequency of home visits and the duration of separation from spouses were found to be closely related to the incidence of risky and unsafe sexual behaviour.

‘Migration and HIV research in Sub-Saharan Africa has focused on HIV risks to male migrants, yet women's levels of participation in internal migration have met or exceeded those of men in the region’ (Camlin *et al.* 2014:146). Migration and HIV research has also focused narrowly on epidemiological concerns and on male migrants, while the role of women has remained obscure (Hunter 2010). Old models such as the male-migration or male-migrant infector model have been used to explain the scale of AIDS and to illustrate contemporary sexual practices (Hunter 2007; Stillwaggon 2006). It is important to focus on other vulnerable groups, such as both male and female migrant workers, if successful and appropriate strategies for intervention in the prevalence of HIV are to be developed.

A correlation between migration and a high risk of HIV infection has been found among rural–urban migrants, and this has been observed among both men and women (Weine & Kashuba 2012). The social processes through which migration contributes to HIV risk are said to be different for the two genders (Camlin, Hosegood, Newell, McGrath, Barnighausen & Snow 2010). Although men are said to gain more from rural to urban migration than women, the migration of women has continued to increase, despite the low earnings to be had in the urban areas (Agesa 2003). In addition, migrant females are fast becoming essential contributors to livelihoods of the poorest households in Sub-Saharan Africa (Collinson, Gerritsen, Clark, Kahn & Tollman 2009). Migration, both internal and external, can also be attributed to volatile economies, rising unemployment, rapid urbanisation and the fall and unsustainable nature of remittances which have led to what Camlin *et al.* (2014:147) describe as the ‘renegotiation of gendered rights, responsibilities and power within households undergoing changes in livelihoods, sometimes leading to marital dissolution’.

It is suggested that apart from migrating to urban areas, as men do in search of economic opportunities and better standards of living, women also seek autonomy and an escape from the often conservative, patriarchal social norms of rural villages (Lippman, Pulerwitz, Chinaglia, Hubbard, Reingold & Diaz 2007). Similarly, the tendency among migrants to engage in risky sexual behaviour has also been attributed to a psychological predisposition towards risk-taking (Brockerhoff & Biddlecom 1999) as a result of disrupted lives (Vissers *et al.* 2008) and exposure to new social environments (Lippman *et al.* 2007). From this perspective, the migrants may be seen to be following modern norms and trends, which are characterised by more egalitarian gender relations. According to Vissers *et al.* (2008), having multiple households may contribute towards involvement with multiple sexual partners or lovers.

In urban areas migrants are exposed to sexual networks having different levels of HIV prevalence and different probabilities of exposure to the pandemic (Hirsch 2014; Hunter 2010). Studies have indicated that the disruption of social networks upon migration might heighten risky behaviour, owing to both diminished supervision and censure and to equally diminished social networks in their new environment, which result in isolation and loneliness (Greif & Dodoo 2011; Hunter 2010). In rural areas there are strong and cohesive social networks and traditional norms, which are respected by all and from which any deviation will attract censure. Townsend, Giorgio, Zembe, Cheyip and Mathews (2014) and Weine and Kashuba (2012) have also successfully established the link between foreign migrants and the increased risk of HIV infection. Similarly, Camlin *et al.* (2010) and Essuon, Simmons, Stephens, Richter, Lindley and Braithwaite (2009) have also documented HIV among migrants in Southern Africa. Although there have been numerous studies focusing on migration and the spread of HIV and AIDS, no studies have been conducted in the industry to establish the link between empowerment and sexual behaviour among migrant workers. Studies of this nature are necessary for the planning of preventive, care and treatment programmes as a component to the national response to the pandemic, with its dire implications for both the industry and the country's economy. It is also essential to continue to monitor high-risk behaviour among migrant populations in order to provide an early warning system.

The migrants referred to in this paper are internal migrants who have moved from the rural areas to the urban areas and the towns, in search of economic opportunities, and who have found employment in the textile industry. The aim of this study was to examine the relationship of economic empowerment and the sexual behaviour of migrant workers within the context of HIV and AIDS, given the fact that most of the workers migrate from the rural areas of Lesotho to the urban areas in order to find employment in the industry. With this aim and on the basis of this analysis, the study sought to find answers to the following research questions: What are the experiences of opportunities for economic empowerment for textile workers and how is their ability to make choices affected by them? What are the social and environmental factors that increase vulnerability to HIV infection in the industry? What is the perception of the workers, regarding changes in their sexual behaviour, as a result of being employed in the industry?

## 2. Research methodology

This paper made use of a larger study, which examined the impact of HIV and AIDS on the textile industry in Lesotho, in the context of the global economic and financial crises. The broader study employed both quantitative and qualitative methodologies to determine the performance of the textile companies, economic empowerment, the vulnerability of women and men to HIV and AIDS and the policies and responses of the textile companies regarding the pandemic in the context of the global economic and financial crises. The researchers came from various disciplinary backgrounds: economics, social work, development studies, demography and statistics.

### 2.1. Research design

Although the broader study employed both quantitative and qualitative methodologies, this paper adopted a qualitative research design located within the interpretative paradigm and focuses on the perceptions of migrant factory workers of economic empowerment, their vulnerability to HIV and AIDS and their sexual practices and behaviour. The rationale for choosing a qualitative approach for this paper was provided by the aim of obtaining a comprehensive description and elucidation of the actual experiences of migrant workers within the textile industry, in line with the approach to research advocated by Dahlberg*,* Dahlberg and Nyström (2008): ‘The primary goal of a basic qualitative study is to uncover and interpret meaning constructed by people’ (Merriam 2009:24). The importance of locating the qualitative approach within an interpretive paradigm lies in the fact that this paradigm views the world as having multiple realities, which may be observed through evidence detailed by those who inhabit the setting (Nieuwenhuis 2007). By using an interpretive paradigm, efforts are made to understand the subjective experiences and realities of the people selected for the study and, by so doing, to retain the integrity of the phenomena being investigated. According to Kieffer (1984) and Maton and Salem (1995) and cited in Sadan (2004:771), measuring the processes of change in the social, physical, political and economic contexts of empowerment ‘ideally requires the inclusion of qualitative research methods’. In addition, Sadan (2004:786) maintains that ‘an empowerment agenda would seem to fit well into this more qualitative or anecdotal approach’. This paper focuses on understanding the shared experiences of textile workers regarding their perceptions of economic empowerment, vulnerability to HIV and AIDS and sexual practices and behaviour. We interpreted and reflected upon the responses of the workers whom we interviewed from the perspective of an ‘outsider’, as is advocated by Cohen, Manion and Morrison (2011).

### 2.2. Selection of participants

The sites used in the larger study comprised all of the 38 textile factories located in Maseru, Mafikeng and Leribe. In order to obtain information which would enhance the selection of an adequate and appropriate sample frame of the factories which would suit the aim of the study, a rapid appraisal questionnaire was administered to the managers of all 38 of the factories. This led to the selection of six factories, five in Maseru and one in Leribe, and a sample of 640 workers was selected to create an equal proportional sample of the workers in each of the selected factories. The sample size for the in-depth interviews was selected from the factories located in Maseru, the capital of Lesotho. Of the 49 textile factories in Lesotho, 24 are located in Maseru, and the Maseru district was purposively selected, as it has more factories than the other districts. The five factories were randomly selected after the collection and analysis of the data from the appraisal questionnaire which had been administered in the 24 factories in Maseru.

A sample of 40 participants was selected for the qualitative component of the study, from which this paper draws its findings. The 40 participants were made up of 20 men and 20 women. These participants were selected from a pool of respondents, who were identified and earmarked by research assistants at the time of administering the questionnaires to the factory workers. The criteria for inclusion in the in-depth interviews were that the worker must have participated in the workers’ questionnaire, works in any of the five selected factories in Maseru and had migrated from the rural areas to Maseru. Eight workers, four females and four males, from each of the randomly selected factories were interviewed. Saturation point was reached as we approached the target number of 40 participants.

### 2.3. Generation of data

The researchers developed a semi-structured in-depth interview guide to gain insights into the shared experiences of the migrant factory workers, in order to understand their perceptions of their economic empowerment, their vulnerability to HIV and AIDS and their sexual practices and behaviour. In-depth interviews constitute an important means by which information pertaining to opinions, personal experience and perceptions may be extracted. According to Creswell (2014), other methods of collecting data are unlikely to yield the same depth of information. The questions which were asked were based on the workers’ perceptions of their vulnerability to HIV and AIDS, their economic empowerment and their sexual behaviour and practices (See Appendix 3 for the in-depth interview guide). The participants responded to these questions during one-on-one interviews with research assistants. Female participants were interviewed by female research assistants, while male participants were interviewed by male research assistants. This was done to enable the participants to express themselves freely during the interviews without fear of gender bias and on account of the sensitive nature of the topic on which the questions centred. The in-depth interviews were conducted in Sesotho, and each was approximately one hour in duration. The interviews were recorded and transcribed for subsequent analysis. During the interviews the participants described what they considered to be relevant shared experiences of economic empowerment, vulnerability to HIV and AIDS and sexual practices and behaviour. These descriptions were interpreted in order to obtain an understanding of their shared experiences and the meanings which they had for them. As had been the case with the questionnaire which had been administered to 640 workers, the in-depth interviews were translated into English for the purpose of writing reports.

### 2.4. Analysis of data

The recorded interviews were transcribed by the same research assistants who had conducted the interviews. This was followed by active and sustained reflection on the responses of the participants concerning their perceptions of economic empowerment, vulnerability to HIV and AIDS and experiences pertaining to their sexual practices and behaviour. The transcriptions were subjected to in-depth analysis by carefully reading the text in order to identify categories and themes, inductively and systematically. The next step was to describe the significant components and recurrent themes which emerged. The descriptions from which the various themes were identified were synthesised in order to be able to substantiate statements by means of direct quotations from the responses given by the participants during the interviews. We allocated codes to all the participants in order to conceal their true identities. The male participants were coded as M1–M20, while the female participants were assigned F1–F20 codes.

### 2.5. Trustworthiness

The trustworthiness of the findings was established as a result of the integrity of the data, which was achieved by obtaining a balance between reflecting objectively on the data on the part of the researchers and the subjective responses of the participants in order to provide a clear communication of the findings in the manner recommended by Williams and Morrow (2009). The adequacy or dependability of the data determines their integrity. First, we ensured that there was balance among the participants by selecting 20 males and 20 females in order to obtain a diversity of viewpoints from the workers who had migrated from the rural mountainous villages to Maseru, the capital of Lesotho. We also ensured that our interpretations correlated accurately with the data by including direct quotations from the verbatim transcriptions of the audio recordings of the interviews with the participants. We also engaged in active and sustained reflection while reading and analysing the data. This enabled us to strike a balance between the responses of the participants, on one hand, and the meanings which we interpreted from them, on the other. In addition, to ensure that objective reflection and subjectivity were balanced, our individual interpretations and those of the research team were used when they seemed most appropriate. Finally, to ensure the clear and coherent development of the paper, we constantly exchanged our written contributions, which provided an additional critical component to the contribution of each and enabled the paper to develop logically. Throughout the writing process, we constantly referred to the research questions in order to ensure that our claims were adequately supported by evidence.

## 3. Ethical considerations

Ethical requirements, such as confidentiality, informed consent and the anonymity of the participants, were strictly adhered to during both the collecting of the data and the analysis and dissemination of the findings. As the National University of Lesotho does not have an Ethics Review Committee, it was left to the researcher's goodwill and conscience to guide the research process in the country. However, the Ministry of Health and Social Welfare provides ethical clearance for medical research, and it had approved the larger study of HIV and AIDS in the textile industry in Lesotho, from which the data for this paper were drawn. See Appendices 1 and 2, respectively, for the letter of introduction and the informed consent form which the participants were required to sign.

## 4. Results

After preparing the narratives, we used Kabeer's empowerment model (2001) and Malhotra's comprehensive framework of empowerment (2003) as the theoretical lenses through which to explore the link between economic empowerment and sexual behaviour in the experiences of migrant textile workers in Lesotho. In other words, we developed themes inductively and pointed to the links between the shared experiences of the workers using these theoretical lenses. The results are presented according to the demographic characteristics of the participants and the following themes: opportunities for economic empowerment, freedom of sexual behaviour and workers’ vulnerability to HIV and AIDS. These themes are discussed by drawing on direct quotations from the participants.

### 4.1. Demographic characteristics of participants

These characteristics of the participants include gender, age range, marital status, educational level, district of origin, duration of working in the industry and the type of contract in terms of which they are employed in the industry. These demographic characteristics are shown in Table 2. Although the industry is dominated by women, an equal number of male and female participants were purposefully selected for reasons of comparison in a research sample comprising 20 male and 20 female workers. Although most of the participants (19) were in the age range of 26–35 years, this group was closely followed by two other sexually active age groups, those from 36 to 45 years old and those from 15 to 25 years old. Of the 40 participants in the sample, there were 13 who had never been married. Most of the participants, 21 in total, had been educated in primary school only, which explained why almost all of them occupied contractually non-permanent positions and only a few were in clerical and management positions (Table 2).

**Table 2.** Demographic characteristics of participants.

Variable	Frequency (numbers only)
*Gender*	
Male	20
Female	20
Total	40
*Age*	
15–25 years	7
26–35	19
36–45	8
46–55	6
56 and above	0
Total	40
*Marital status*	
Never married	13
Currently married	11
Divorced/separated	2
Widowed	9
Cohabitation	5
Total	40
*Education*	
Never attended school	5
Primary [PLSC]	21
Junior secondary [JC]	6
Senior secondary [COSC]	8
Tertiary education diploma	0
Vocational training	0
University degree	0
Total	40
*District of origin*	
Leribe	3
Botha Bothe	5
Berea	2
Mokhotlong	2
Maseru	10
Thaba Tseka	4
Mohale's Hoek	6
Mafeteng	4
Quthing	2
Qacha's Nek	2
Total	40
*Duration of employment*	
0–5 years	4
6–10 years	20
11–15 years	11
16 years and above	5
Total	40
*Type of contract*	
Permanent contract	5
Temporary contract	23
Casual/daily work	12
Other	0
Total	40

The participants were from all 10 districts of the country and this selection was made deliberately in order to have a sample which was a fair representation of the entire country. Maseru, the capital city with a huge population, was represented by 10 participants who had migrated to the city. The rest of the districts shown in Table 1 were represented by between two and six participants each. Twenty of the participants had worked in the industry for between 6 and 10 years, 11 had worked from 11 to 15 years and the rest had worked either from between 0 and 5 years or else for 16 years or more. Where the type of contract held by the participants was concerned, most of them (23 participants) had temporary contracts, and only 5 had permanent contracts.

### 4.2. Many opportunities for economic empowerment

Considering the poverty that rural–urban migrants face in many cities, especially in Africa, one of the main themes that emerged from the discussions was the wealth of opportunities for economic empowerment provided by the industry. First, all of the participants indicated that they were able to provide for their individual and household material needs as a result of earning a salary from working in the industry. They all reported that when they were unemployed, they could not afford the basic necessities of life: food, shelter and clothing. The participants maintained that employment in the industry had given them the opportunity to be able to acquire goods such as groceries, clothes and household items, and that they were able to pay school fees and buy school materials for their children and other relatives. Accordingly, most of the participants reported that life had improved significantly for them and their family members. The following excerpts from some of the interviews highlight their appreciation of what employment in the textile industry has done for them as a result of earning a salary which has economically empowered them:

Now my life has changed because I am able to buy my children's school uniforms, books, shoes and other needs. I am able to look after myself and my family back at home. (F17)

I can now get some of my basic needs with ease unlike before, though I am still struggling since the income is small. ‘Half a loaf of bread is better than none’. I can proudly say that I am living a decent life. (M2)

I am able to build a small house for my family and this gives me satisfaction as a man who can provide for his family. My family is proud of me because of who I am as a result of being employed in this factory. (M13)

However, 10 of the female participants claimed that the money which they earned was not enough to meet their needs. A female participant said: ‘Though I am working and earning a salary, every month, I need additional money to meet my household needs, such as clothes, food and transport’ (F19). Another disgruntled worker said: ‘The salary is too small to have a significant impact on my life, especially with our African dependents that one needs to cater for’ (F15). These women reported that employment in the textile industry had not brought any meaningful change in their lives, citing the skyrocketing prices of basic commodities, resulting from the global financial crisis of 2007 and 2008, as the reason for this. Not only were some of the female workers dissatisfied with the meagre salary they earn in the industry, but this was also the case for some of the male workers. Four men who had been retrenched from the South African mines some years previously and were currently employed in the Lesotho textile industry expressed dissatisfaction with their salaries by comparing them with what they had previously been earning as mineworkers in South Africa.

Second, the overwhelming majority of the participants, both male and female and 37 in total, agreed that earning a salary had given them an opportunity to experience increased options and choices in their lives, and the ability to make independent choices. They reported that being employed in the textile industry had enabled them to be able to make important choices, which included choices regarding how and when they spent their money and what they spent it on. A female participant said:

Now that I have my salary, I decide on how and on what to spend money, without consulting anyone; neither am I restricted on what things I need to spend my money on. (F2)

Demonstrating awareness and an understanding of modern perceptions of rights and reciprocity, one of the male participants said:

I make choices and decisions with my wife on how and on what to spend my money on. My wife is my equal partner, so I always consult her on everything. (M12)

However, a few of the participants maintained that there had been no change in the decision-making process in their households, as they had been the sole decision-makers in their lives. This type of response tended to come from most of the single participants, both female and male, who were not in serious relationships and involved with casual sexual partners only. Finally, all of the female participants agreed that as a result of employment in the textile industry they enjoyed a degree of self-confidence which they had not experienced before. Some of them cited the fact that there are no discriminatory laws in Lesotho against either gender, and that neither are there discriminatory laws in the industry. In the light of this, they maintained that gender equality was respected in their workplaces, apart from the fact that many of them held unskilled labouring positions in the industry owing to their low levels of education. One of the female participants said:

Employment has given me confidence as a woman. Being able to bring my salary home at the end of the month really makes me proud as a woman. This makes me feel equal to my husband because we both bring something home at the end of the month. (F5)

Another of the female participants summed up the issue of gender equality with these words:

As a woman, I feel confident and empowered due to my employment. I feel women are treated equally to men. We do not feel unfairly treated. (F1)

### 4.3. Freedom of sexual behaviour and practices

A question which might be asked is whether working and earning a salary as a migrant worker promote freedom of sexual behaviour and practices. The responses of 36 of the participants, when questioned in relation to this theme, indicated that employment and earning a salary in the textile industry in Lesotho give workers the freedom to be able to make various choices about important issues such as sex with or without condom, with whom and when to have sex. Of these 36 participants, the majority (32) were women. One of them proudly revealed:

I am now very comfortable and confident at all times because no man can instruct me; hence my ego is boosted as a woman who takes my destiny into my hands, especially with my sexual life. (F11)

For most of the male participants (15 of the males), these issues had been the exclusive domain of males, although for them employment in the industry has simply reinforced and increased these privileges and rights in sex-related issues. For the female workers, however, working and earning a salary have empowered them economically to be able to care for their families without necessarily asking their husbands or boyfriends for financial assistance, giving them the freedom to engage in whatever sexual behaviour and practices they might desire. Nonetheless, two female participants disagreed that their employment in the sector had enabled them to make different choices regarding sexual matters. A female participant maintained that, as a married woman, she had always had to defer to her husband to make such important decisions and choices for her, irrespective of her employment status. She went on to explain that the respect for their customs and traditions, which was demanded, prohibited women from deciding on sex-related issues.

The responses of five participants led to the emergence of expansion of sexual relationships as a theme relating to the main theme. They reported that earning a salary in the textile industry enabled them to have more sexual partners, to socialise with friends of the opposite sex and also to be able to enjoy a ‘variety’ of women. A young worker, excited by earning a salary after a long period of unemployment, revealed that it was the right time for him to engage in sexual activities since he now had the money to play and to enjoy life. According to him:

I make decisions on what to do, especially with my sexual life. I now engage in sexual relationships with many girls now that I am earning a steady salary, whereas before I could not because of poverty. (M14)

On the other hand, two male participants explained that they had decided to limit their sexual behaviour. The main reason which they gave for this was the desire to be respected now that they earned a salary and were looked upon as role models in their villages. However, no female participants expressed the need to increase the number of boyfriends or sexual partners they had as a result of employment in the industry. Nonetheless, the fact that several of the men had multiple sexual partners should probably be attributed to the gendered nature of transactional sex and also to expectations for them to be financial providers.

### 4.4. Workers are vulnerable to HIV infection

When the participants were asked where they were staying while their spouses and families were back at home, 37 of them said they were staying near the factories where they worked and only occasionally visited their homes and families when taking leave, and that many of them worked overtime during the weekends and public holidays. When they were asked whether employment in the textile industry and being away from home had increased their vulnerability to HIV infection, the responses of 34 of the participants, in relation to this theme, indicated that there is an increased likelihood of being infected with HIV, as most of them were staying far away from their homes and spouses. Among the reasons for this vulnerability advanced by 14 of the male participants were peer pressure and the large numbers of attractive female workers in the various factories. Four of the male participants offered that with money, a lot can ‘happen’, and that, as a result of earning a salary, they were able to have more girlfriends and to enjoy social life with them, which would inevitably be accompanied by risky sexual behaviour.

According to 16 of the female participants, men do not like to use condoms and their unwillingness to be tested for HIV led to increased vulnerability to it, which was detrimental not only for male workers, but also for female workers. Closely related to the main theme are issues pertaining to the practice of transactional sex, which were revealed by a few of the female workers. Owing to the insufficient wages which these workers earn, three female workers confided that they would like to ‘suck’ money from men in exchange for sex, in order to supplement their incomes. These three women said that as men do not always want to use condoms and as the women are desperate for money, they would definitely give in to demands for unprotected sex if necessary. However, among the rest of the participants, six, both male and female, did not agree with this reasoning, while two female participants did not want to be involved in casual relationships because they were faithful to their partners. One of them said:

I am married and if I want sexual satisfaction, my husband is there at home, a few kilometres away. He usually comes around if I am tied up with work. (F8)

The other female worker stressed that there are preventive campaigns and posters which educate and make people aware of the issues related to HIV and AIDS. She cited the campaigns by Apparel Lesotho Alliance to Fight AIDS and the role that they have played to educate the workers, including those already infected with HIV.

## 5. Discussion

It is important to recognise the benefits of empowering rural–urban migrant workers through economic opportunities, especially poor women from the remote mountainous villages. Empowerment has been defined in terms of increasing control over one's life and also of participating in community activities (Lord & Hutchison 1993). The findings of this study show that all the participants felt positively about being employed in the textile industry and earning a salary. The positive response was stronger among women than men, as earning a salary increased the ability of the women to provide basic individual and household needs, to be able to make choices affecting their lives and to participate in household decision-making. As the findings show, employment promotes self-confidence among the workers. This self-confidence needs to be understood from two standpoints: first, Lesotho has a high unemployment rate (45%) (United Nations Statistics Bureau 2010), with most of the unemployed being women. Second, Lesotho is a patriarchal society where the role of women is still, to a very large extent, restricted to procreation and domestic chores.

Employment in the industry has brought a variety of economic opportunities to the workers, who had previously been poverty-stricken, powerless and voiceless. Previously, these migrant workers would almost certainly have seen themselves as being powerless and incapable of having control over their own lives, or of being able to influence other people in their lives (Greif & Dodoo 2011). For these workers earning a salary has placed them in situations where ‘life transitions’ are possible and they are able to build on their inherent strengths and capabilities, which resonates well with two of the five main impetuses for empowerment, described by Lord and Hutchison (1993:10). Accordingly, the participants see themselves as having been personally empowered. The findings show that the textile workers have experienced opportunities for economic empowerment, to a certain extent, in that they now have the power to control their lives, the right to have access to opportunities and resources and the right to have and to determine choices. However, the level of economic empowerment that the workers have acquired cannot enhance their ability to influence the direction of social change that is needed for the creation of social justice and economic order, nationally and internationally, which is one of the five important components of empowerment laid down by the United Nations (UN 2001). In addition, owing to their inability to influence the direction of social change, the degree of participation by the workers in the community could be seen as still being very timid and tentative, affording them less dignity than Lord and Hutchison's (1993) definition of empowerment demands.

The findings show that 14 of the participants (10 women and 4 men) were not happy with the meagre salary that they were earning in the industry. This dissatisfaction stemmed from the fact that although they were employed, they still could not afford enough of the basic necessities to ensure a better standard of living for themselves and their dependents. This could very well explain the reason for some of the participants engaging in transactional sex in order to supplement their incomes and also to increase their ability to buy modern consumer goods. Sixteen of the female participants complained that men do not want to use condoms and are also not willing to be tested for HIV, resulting in some women engaging in risky sexual behaviour for fear of losing their partners (Oliver 2011). Three of the female participants admitted that they engaged in transactional sex. Many recent studies have demonstrated that women's motivations for engaging in transactional sex are aligned to globalisation, in that the proceeds from transactional sexual encounters are used to purchase the symbols of global beauty propagated by the local and global print and visual media (Camlin *et al.* 2010, Greif & Dodoo 2011; Hirsch 2014; Hunter 2010; Jordal, Wijewardena, Öhman, Essen & Olsson 2014; Roy *et al.* 2010; Stoebenau, Stephanie, Rubincam, Willan, Zembe, Tsikoane, *et al.* 2011; Weine & Kashuba 2012; Zembe, Townsend, Thorson & Ekstrom 2013). These women have come to be known as ‘global citizens in local states’ (Robins 2003). It is believed that women with better paid jobs would be able to secure housing independently of men and enter into sexual relationships on their own terms (Stoebenau *et al*. 2011). Owing to low wages, the desire to meet the basic needs of survival and the quest for the consumption of the consumer goods provided by modern technology and modern standards and expectations, these migrant female workers have chosen transactional sex as a means of supplementing their incomes.

However, it has been argued that money is not the only motivating factor for women engaging in sex–money exchanges and that, sometimes, there are other reasons. For example, Hunter (2007) argues that gifts are often given in terms of men's perceived role of provider and on the basis of ‘love’ and ‘physical attraction’, and not in sex–money exchanges. Campbell (1997) found a correlation between social support and risk-taking sexual behaviour among mine workers in South Africa. She concluded that one of the factors driving HIV transmission is limited social support and scant opportunities for intimacy to miners. According to Tawfik and Watkins (2007), women are motivated not only by money, but also by the desire for attractive consumer goods, by passion and sometimes revenge for a husband's infidelity. Accordingly, women should not been seen as powerless and unfortunate victims, used by men to satisfy their sexual desires, but rather as active agents of sexuality. Although there is no evidence in this study which correlates with the findings of these other studies, considerations of this sort are necessary in any analysis of transactional sex.

While some of the female participants in this study engaged in transactional sex as a result of their meagre salaries, five of the male participants increased the numbers of sexual relationships in which they were involved as a result of earning an income from the industry. This thesis is supported by Hunter (2007), who argues that richer men are more able to secure multiple girlfriends than poor ones. Although male workers also have an ‘appetite’ for more money, this can only be achieved through extra work or overtime as there are few ‘sugar’ mummies (Roy *et al.* 2010). Accordingly, the few male workers who have increased their numbers of sexual partners can conclusively be linked to their economic empowerment in the industry. However, this finding cannot be generalised to all or most of the male workers in the industry.

The study found that most of the participants (37 out of 40) were staying far away from their homes and visited their homes only occasionally, as a result of being tied to a work schedule. Consequently, they experience the isolation and loneliness which result from what Greif and Dodoo (2011) call ‘disruption of social networks’. This probably explains why 34 of the participants agreed that working in such an environment is likely to lead to risky sexual behaviour and practices, and this correlates with the views expressed by Vissers *et al*. (2008). There are many social and environmental factors which may increase the need for additional income, including poverty, deprivation and the need to enjoy modern goods such as cell phones, iPads, cameras, facial and body make-up and clothes. Women may be particularly motivated to want to acquire some of these goods, as they are known to be very competitive, and low income is likely to encourage some of them to engage in risky sexual practices in order to meet the material standards achieved by their friends (Townsend, Giorgio, Zembe, Cheyip & Mathews 2014).

## 6. Conclusion

After considering the findings of the study and the discussion that followed, we return to the theoretical framework of empowerment in order to interpret the meanings of the experiences of economic empowerment and the sexual behaviour and practices of the participants. The first two main themes (opportunities for economic empowerment and freedom of sexual behaviour and practices) demonstrate how the theory of empowerment is able to highlight areas in which migrant textile workers are economically empowered and those in which they were still lagging behind. According to Kabeer's (2001) empowerment model and the economic dimension at the household level of Malhotra (2003), the workers were able to afford to choose and provide material alternatives for themselves and their households and also had the ability to make choices and to control household income. However, there was no evidence to suggest that the alternatives created by the workers for themselves and their increased capacity to make choices might result in a transformation of gender relations.

Regarding the ‘power to’ (Kabeer 2001), the workers were able to participate in household decisions, to solve their individual and household problems and to make independent sexual decisions. Consequently, the resulting increased vulnerability to HIV infection is not only the result of their acquiring ‘power to’, but also of environmental factors. ‘Power to’ corresponds to Malhotra's (2003) comprehensive framework dimension of the familial and interpersonal, at the household level. However, the ability of the workers to use power to be ‘creative’ and ‘enabling’ has still not been increased as a result of their being employed in the industry. Where the psychological dimension of Malhotra's framework (2003) at the household level and Kabeer's (2005) empowerment model concerning ‘power within’ are concerned, although the workers may have achieved some degree of self-confidence, they have yet to attain self-awareness and assertiveness or the power to act, influence and change (Oxaal & Baden 1997:1). Although their empowerment has enhanced the workers’ consciousness of their place in the power relations, their achievements are not felt in the existing realm of power. This is almost certainly owing to the fact that the workers do not occupy management positions in the industry as a result of their low educational qualifications and also because they need to act and participate in the industry's activities and community as a collective. Although there is a perceived relationship between economic empowerment and the risk of HIV exposure, there is no strong evidence to suggest that their exposure to risky sexual practices is solely the result of having been economically empowered, and social and environmental factors certainly need to be taken into account. Nonetheless, it is argued that economic empowerment helps to reduce risky sexual practices among those who are vulnerable, owing to deprivation and poverty (Tanga 2013), and the findings do show that the workers have been able to acquire some basic survival needs, such as household goods, groceries and school fees for their dependents, as a result of earning a salary. Some empowerment experts will inevitably disagree that the acquisition of some basic needs can be equated with empowering the workers. However, these perceptions of economic empowerment are skewed, as many of the workers are still burdened by the hardship of not being able to meet their basic household needs.

There is great potential for the empowerment of individual migrant textile workers because empowerment could help them to acquire an understanding of their situation, thereby giving them a new social meaning for it and for their relations with others in the industry. In the long run, empowerment could also lead to economic growth and the achievement of all the Millennium Development Goals. Workers, and women in particular, must become significant actors in the process of change; otherwise, there would be only what Malhotra (2003) calls ‘improvement in the outcomes from one point to another’. The degree of access to necessary resources, such as preventive campaigns, education, treatment and relationships, determines the ability of the workers to act and to influence (Sadan 2004). Important aspects of empowerment for the participants, which need to be considered, include access to essential resources such as education, support from those who wield influence and participation. It is also vital to ensure that empowerment is a systematic transformation, not only at the institutional level, but also in the supporting patriarchal structures (Malhotra 2003). Education has long been used for the purpose of promoting equal opportunity and empowerment. Educational programmes for the benefit of the workers need to be instituted if their economic empowerment is to have any real significance. A better understanding of the migrants and their patterns of risky sexual behaviour could be of great benefit for both local government and healthcare officials. Gender transformative programming needs to encompass economic empowerment approaches, as hegemonic gender roles and inequalities need to be addressed to enable women to take full control of making their own sexual decisions.

### 6.1. Limitations of the study

The study has some limitations which need to be mentioned. First, the results are based on qualitative data obtained without making a cross-sectional survey. The behavioural findings are based on self-reports and, as a result, the accuracy of the data cannot be tested or verified. In addition, individual participants may not have provided completely honest answers, given the sensitive nature of the topic on which the questions were based. Second, the study did not make use of statistical analyses to determine the extent of the relationships between the variables: in this case economic empowerment and sexual practices and behaviour, on one hand, and the extent of economic empowerment, on the other. Third, it is also difficult to assess the degree to which the findings may be relevant to other groups of rural–urban migrants in Lesotho, given the high specificity of the sample of migrant workers. However, it is believed that many of the behaviour patterns found in this study would be found in equal measure among other rural–urban migrant groups in Lesotho. Finally, although several other domains have been added to Kabeer's model of empowerment, this study confined itself to only two: ‘power to’ and ‘power within’. Similarly, only three dimensions of Malhotra's (2003) comprehensive empowerment framework at the household level were considered for this study: the economic, the familial and interpersonal. However, the use of these two frameworks provided much useful data for an individual analysis of empowerment.

## Appendix 1: Letter of introduction

### ECONOMIC GLOBALISATION – HIV AND AIDS – NEXUS IN THE TEXTILE *and* GARMENT INDUSTRY IN LESOTHO

- The National University of Lesotho – in collaboration with IDRC/HEARD is undertaking a research on Economic globalization–HIV/AIDS nexus in the textiles and garment industry in Lesotho in which you have been selected to participate. The study will investigate the interconnections between economic globalization and HIV/AIDS in this industry.
- There are no known direct benefits associated with your participation in this research. However, the data will enable us to provide the stakeholders, including industry and policy makers, with a basis for inform decision regarding policy that may lead to overall improvement in the sector.
- You will be asked various questions by a trained interviewer relating to your economic situation, empowerment and gender vulnerability some of which may be sensitive but important for the success of this effort. This interview will take approximately **1 hour** to complete. It is important to note that the answers to all these questions will remain confidential neither you nor your factory will be identified by name. Anonymity will be maintained throughout the study and dissemination process. The sheet with your answers and the accompanying signed consent form will be securely kept for some period of time pending when they will finally be destroyed.
- Your participation is entirely voluntary and a national service. You are however free to decline to answer any specific question if you feel the information is too sensitive or personal. You are also at liberty to tell your interviewer that you no longer wish to participate in the interview and your responses should not be used for the research so that the answer sheet with your consent form will be destroyed with no consequences. Should you agree to participate, you need to sign this informed consent form. You are free to refuse to participate in the study if you wish.

Should you require any additional information concerning this study, you are welcome to contact any of the following persons:

**NB: Names and contact numbers withheld for purpose of anonymise review**

## Appendix 3: In-depth interview guide for factory workers

Respondent/Participant ID Number: _______ Firm ID Number: ___________

### Section A: Socio-economic information

Q1. What is your employment status (e.g. permanent; casual; temporary)?
Q2. What is your level of responsibility (e.g.Manager; supervisor; ordinary worker)?
Q3. How long have you been working for this firm?
Q4. What is your age?
Q5. What is your educational level?
Q6. Sex of respondent

### Section B: Vulnerability

Q7. Since beginning work at the factory do you still require additional money in order to meet your needs? (Yes or No)
Q8. If Yes to Q7 can you give examples of such needs? (please ask for a lot of specific details here)
Q9. If so, how have you obtained money to meet such needs?
Q10. Since becoming employed at the factory have you begun a relationship or had sex with someone because she/he provided you with, or you expected that she/he would provide you with, any of the following: food, cosmetics, clothes, cell-phone, transport, school fees, money for rent, and money for tuition, somewhere to sleep or cash?
  If yes please provide some more information. In the last year how many different partners like this have you had? How often do you have such sex (daily/weekly/monthly/once a year)? And which items did you expect or receive? please list them.
Q11. Before you became employed at the factory did you ever had sex in the hope of receiving any of those items? (For probing, e.g. food, cosmetics, clothes, cell-phone, transport, school fees, money for rent, and money for tuition, somewhere to sleep or cash?
  If yes please provide some more information. How many different partners did you have? When did this occur? How often did you have such sex (daily/weekly/monthly/once a year)? And which items did you expect to receive or actually receive? Please list them.
Q12. Describe in which particular places you have stayed or lived since birth for more than six months at a time.
Q13. Do you think life in Maseru/Leribe is different to where you come from? Yes/No
  - If so, how?
  - Has it changed your friendships?
  - Has it changed your sexual relationships?
Q14. Have you ever had sex even though you knew that this may put you at risk of contracting HIV? And please also tell me why you felt you were at risk i.e. – no condom was used; you knew your partner has multiple/many partners, the sex was violent, you knew your partner was HIV positive etc. If so can you tell me why you were not able to avoid the sexual encounter?
Q15. Do you know of anyone who had sex with a co-worker or boss because he/she felt he/she had to in order to keep his/her job or get a promotion? If so please tell me about the situation.
Q16. Do you think you might be at risk of becoming infected with HIV? YES/NO If so why?
Q17. Do you think the workplace increases your likelihood of contracting HIV? YES/NO If so why?
Q18. (If they answered Yes to Q16) If you think you might be at risk of contracting HIV what do you do to reduce your risk?
Q19. Do you think men and women who are HIV positive are discriminated against in the workplace any way? [YES/NO] If so please, explain what happens.
Q20. In your workplace do they offer the following (answer Yes/No):
  - Provide you with training about HIV and AIDS?
  - Provide condoms
  - Provide HIV voluntary testing and counseling?
  - Provide treatment for HIV?
Q21. If yes to any of Q20, do you use the services? And why, or why not? (Please ask for specific details for each “Yes” answer in Q20 a–d)

### Section C: Empowerment

Q22. How has earning a salary through employment in the factory increased your options and choices? If you were unemployed, or earned a lower salary, before please tell us what has changed for you now that you have a salary.
Q23. Do you think that unemployment would affect your ability to make choices about important issues concerning sex. Such as when to have sex, who you have sex with and whether to use a condom etc (Yes or No)? Please explain why, in detail.
Q24. Has employment in the factory enabled you to make different choices about important issues, such as:
  - Who decides how the money you earn is spent? Can you now make those decisions?
  - What the money you earn is spent on?
  - Please tell us in detail what you think about this change?
Q25. Has employment in the factory enabled you to make different choices about important issues concerning sex? Can you now make decisions about issues such as:
  - whether to have safe sex (using condoms etc)?
  - who to have sex with?
  - when you have sex?
  - explain in detail what this change has meant for you.
Q26. If you should lose your job, how will this impact on any POWER that you currently have over making CHOICES in your life? This could be power and choices about how to spend your money and/or power that you have in your personal relationships.
  In particular would losing your job affect your ability to make key decisions about **when to have sex**, **who to have sex with** and **whether you could protect yourself from HIV infection during sex**?
  If you think you may lose power please explain why.
  Please use examples to help explain how some of the decisions you make may have changed or not.
Q27. **(For women only)** Do you feel that being employed in the factory has made you feel more confident as a woman? Do you feel that you have become more aware of your right to be treated equally to a man, and if so please give us an example to explain this? However, if you feel you are still not treated equally to men please tell us why you think this is the case, and give us an example of how you are treated differently.

**Thank you for your time and participation**
